# First report of the presence of *Acartia bispinosa* Carl, 1907 (Copepoda, Calanoida) in a semi-enclosed Bay (Sharm El-Maya), northern Red Sea with some notes on its seasonal variation in abundance and body size

**DOI:** 10.3897/zookeys.444.7633

**Published:** 2014-10-08

**Authors:** Mohsen M. El-Sherbiny, Ali M. Al-Aidaroos

**Affiliations:** 1 Department of Marine Biology, King Abdulaziz University, Jeddah 21589, Saudi Arabia; 2 Department of Marine Sciences, Suez Canal University, Ismailia 41522, Egypt

**Keywords:** Copepods, *Acartia bispinosa*, morphology, seasonal abundance, body size, Red Sea

## Abstract

The calanoid copepod, *Acartia bispinosa* Carl, 1907, is reported for the first time in the Red Sea, where it is found to be an important copepod in the mesozooplankton community structure of the Sharm El-Maya Bay. Female and male are fully redescribed and illustrated of as the mouthparts of this species have never previously been described and figured. *Acartia bispinosa* was collected in the plankton samples throughout the year and showed two peaks of abundance, a pronounced one in April (4234 individuals m^-3^), and second smaller peak during November (1784 individuals m^-3^). The average total length of females varied between 1.32 and 1.53 mm at the end of June and January respectively. For males, the average total length fluctuated between 1.07 and 1.16 mm at end of June and March respectively. Temperature showed an inverse relationship with the body length (*P* > 0.001) and seemed to be one of the prime factors affecting the body length of both sexes.

## Introduction

Acartiidae Sars, 1903 is a speciose family of copepods, that inhabits estuarine and neritic environments all over the world from boreal to tropical regions (Bradford 1976, Mauchline 1998, Belmonte and Potenza 2001). They are thought to be mainly adapted to high food concentrations, which are encountered in estuaries and upwelling regions. They are key organisms to link between the primary producers and secondary consumers in many neritic and inlet waters (Putland and Iverson 2007), since they are the major consumers for phytoplankton and microzooplankton and serve as a prey for fish larvae (Seki and Shimizu 1997). A number of *Acartia* species produce diapause eggs, which allow them to lie dormant in the sediment and to appear in the plankton when the conditions are favorable (e.g. Engel 2005).

So far, nine species of *Acartia* have been recorded from the Red Sea (Halim 1969, Razouls et al. 2014), namely, Acartia (Acanthacartia) fossae Gurney, 1927, Acartia (Acartia) danae Giesbrecht, 1889, Acartia (Acartia) negligens Dana, 1849, Acartia (Acartiura) clausi Giesbrecht, 1889, Acartia (Acartiura) longiremis (Lilljeborg, 1853), Acartia (Odontacartia) centrura Giesbrecht, 1889, Acartia (Odontacartia) erythraea Giesbrecht, 1889, *Acartia eremeevi* Pavlova & Shmeleva, 2010 and *Acartia mollicula* Pavlova & Shmeleva, 2010. During a year round study of the planktonic copepods in the coastal waters of a semi-enclosed bay near Sharm El-Sheikh, northern Red Sea, four species of *Acartia* (*Acartia centrura*, *Acartia fossae*, *Acartia danae* and *Acartia negligens*) were collected, in addition to a newly recorded species to the Red Sea (Acartia (Odontacartia) bispinosa Carl, 1907), which was found to be a dominant species in our samples. In this paper, *Acartia bispinosa* is redescribed and illustrated, since the original description is limited as well as the mouthparts of this species have never previously been described. Also, the seasonal variations in their abundance and body size in relation to the different environmental parameters were discussed.

## Materials and methods

*Acartia bispinosa* specimens were collected at monthly intervals from Sharm El-Maya Bay (with an average depth of 3 m), in the northern Red Sea (27°51'36"N and 34°17'39"E) using a conical 0.1 mm mesh plankton net (mouth diameter of 0.4 m and total length of 160 cm) fitted with a Hydro-Bios flowmeter, from January to December 2009. The net was towed horizontally for ten minutes 0.5 m beneath the sea surface and filtered volume was estimated from the flowmeter reading and the net diameter. Immediately after collection, samples were fixed in a final concentration of 4% formaldehyde-seawater until analyses after several months. According to Durbin and Durbin (1978), the period of preservation has no significant effect on the length of *Acartia*. From each sample, 50 specimens of both adult females and males of *Acartia bispinosa* were measured for the total length and the prosome length using a Nikon stereomicroscope (205A) equipped with the software LAS (Leica Application Suite). Microscopic examinations and dissections were made in lactophenol using bright-field and differential interference microscopes (Nikon E600). For detailed observation, specimens were stained with a 0.1% chlorazol-black E solution. Drawings were made with a camera lucida. Terminology follows Huys and Boxshall (1991). For scanning electronic microscopy (SEM), whole copepods or the dissected parts were mounted on stubs, dehydrated with liquid nitrogen, coated with white gold, and examined in a JEOL, JSM-5600LV scanning electron microscope. Temperature, pH, salinity and dissolved oxygen from the surface water were measured using a multiparameter water quality meter (Horiba U-50). For chlorophyll *a*, five liters of surface water were collected and filtered through 35 mm diameter Sartorius membrane filters (pore size 0.45 µm), extracted in 90% acetone and analyzed spectrophotometrically following Parsons et al. (1984). The Pearson correlation coeﬃcient at a confidence limit of 95% was applied to study the relationship between the abundance of *Acartia* and the other environmental factors. Also, ANOVA was used in order to test if differences between months regarding body size were significant or not. All these statistical analyses were performed with the help of SPSS software (Version 16).

## Results

### Description

#### Order Calanoida G. O. Sars, 1902 Superfamily: Diaptomoidea Baird, 1850 Family: Acartiidae Sars, 1903 Genus: *Acartia* Dana, 1846

##### Acartia (Odontacartia) bispinosa

Taxon classificationAnimaliaCalanoidaAcartiidae

Carl, 1907

Acartia amboinensis (F): Sewell 1914 a (p. 242, figs 1–7 Only female)
Acartia tokiokai Mori 1942 (p. 556, pl. 11, figs 1–18)
Acartia hamata C.B. Wilson 1950 (p. 152, figs 1–5)

###### Description.

**Figure 1.**
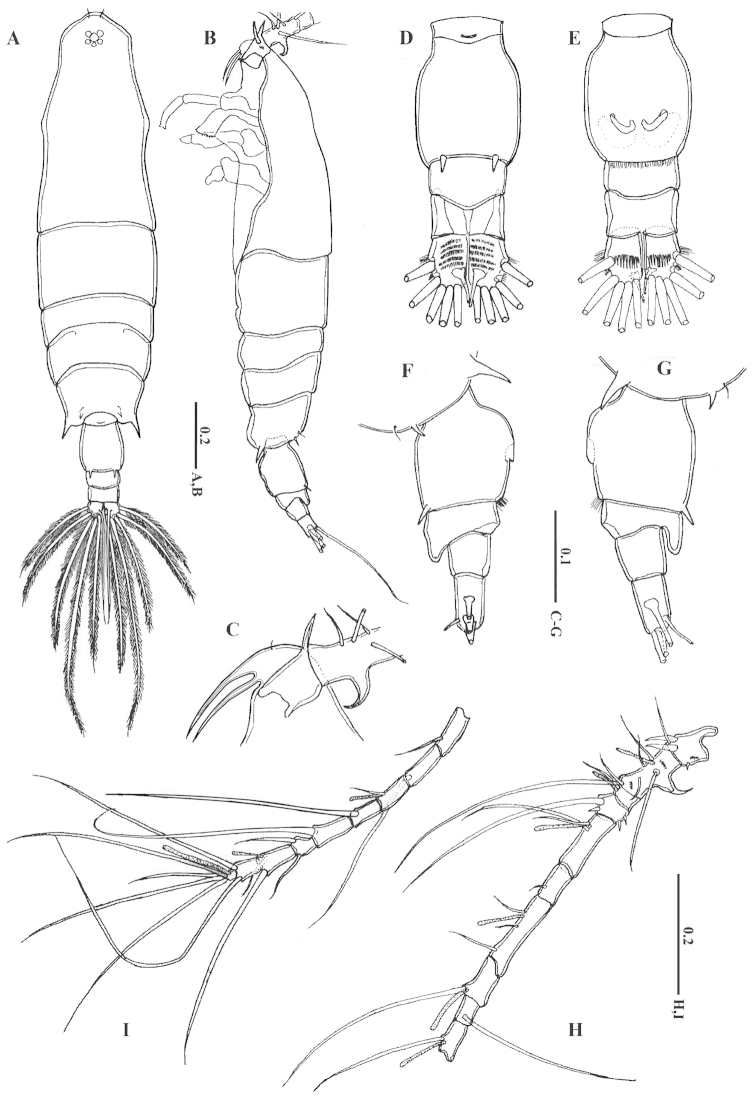
*Acartia bispinosa* female from the northern Red Sea. **A** habitus, dorsal view **B** habitus, lateral view **C** rostrum and proximal part of the antennule, lateral view **D** urosome, dorsal view **E** urosome, ventral view F urosome, later view right **G** urosome, lateral view left **H–I** antennule. All scale bars in mm.

**Figure 2.**
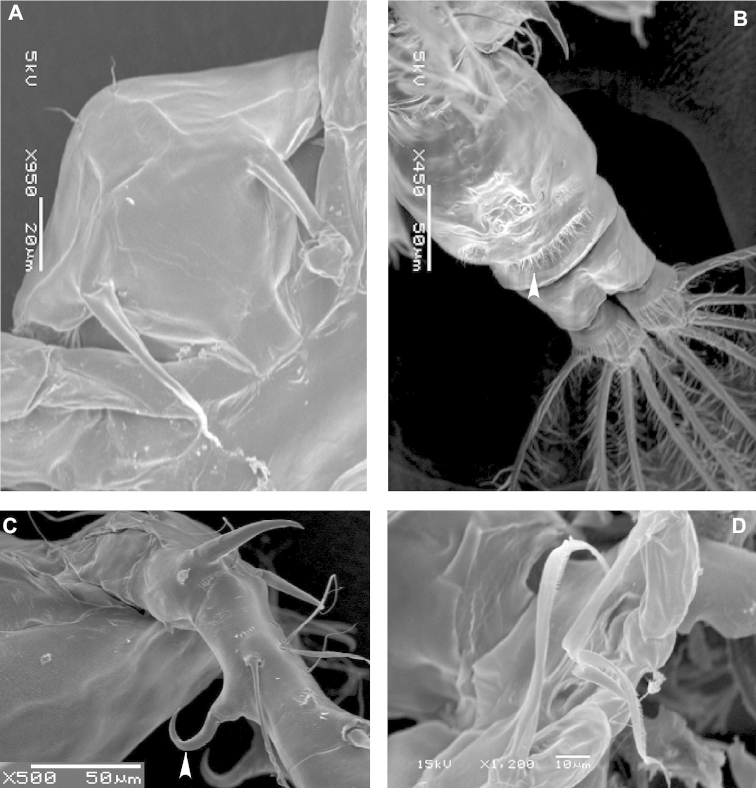
SEM micrographs of *Acartia bispinosa* female from the northern Red Sea. **A** rostrum, ventral view **B** urosome, fine hairs on the posterior margin of genital compound somite indicated by arrow, ventral view **C** proximal part of antennule, claw-like curved spine indicated by arrow, lateral view **D** Female leg 5.

Female: Body (Fig. 1A, B) slender; prosome 5-segmented; rostrum (Fig. 1C) with two long rostral filaments and paired frontal sensilla (Figs 1C, 2A). Nauplius eye present. Cephalosome and first pediger completely separate; fourth and fifth pediger fused; fifth pediger with lateral strong projection, posterodorsal spine and fine setule on each side (Fig. 1A, F–G). Urosome 3-segmented; genital compound somite (Fig. 1D–G) slightly longer than wide, carrying two spines postero-dorsally reaching nearly one-third of second urosomite, posteroventral margin furnished with very fine hairs (Figs 1E, 2B); second and third segments naked. Caudal rami symmetrical; with 5 transverse rows of fine setules dorsally and 1 row anteroventrally (Fig. 1D–E), each ramus bearing six setae.

Antennule (Fig. 1H, I): incompletely 19-segmented; second to third and fourth to sixth segments partly fused on dorsal surface; segmentation and setation patterns as follows (SS specialized spine): (1) I=1+2SS, (2) II–VI=6+SS+1 aesthetasc (ae), (3) VII–VIII=2, (4) IX–X=2 (1 spiniform), (5) XI–XII=2+ae, (6) XIII=1, (7) XIV–XV=3+ae, (8) XVI=1+ae, (9) XVII=1, (10) XVIII=1+ae, (11) XIX=1, (12) XX=1, (13) XXI=1+ae, (14) XXII=1, (15) XXIII=1, (16) XXIV=1+1, (17) XXV=1+1+ae, (18) XXVI=1+1, (19) XXVII–XXVIII=4+ae. First segment with short spine at distal half and 2–3 spinules on dorsal surface; second segment with strong claw-like spine curved proximally from mid-posterior margin (Figs 1C, 2C), transverse row of 2–3 spinules on proximal half of dorsal surface and transverse row of 4 spinules on distal half of dorsal surface; fourth segment with 2 spines dorsally.

**Figure 3.**
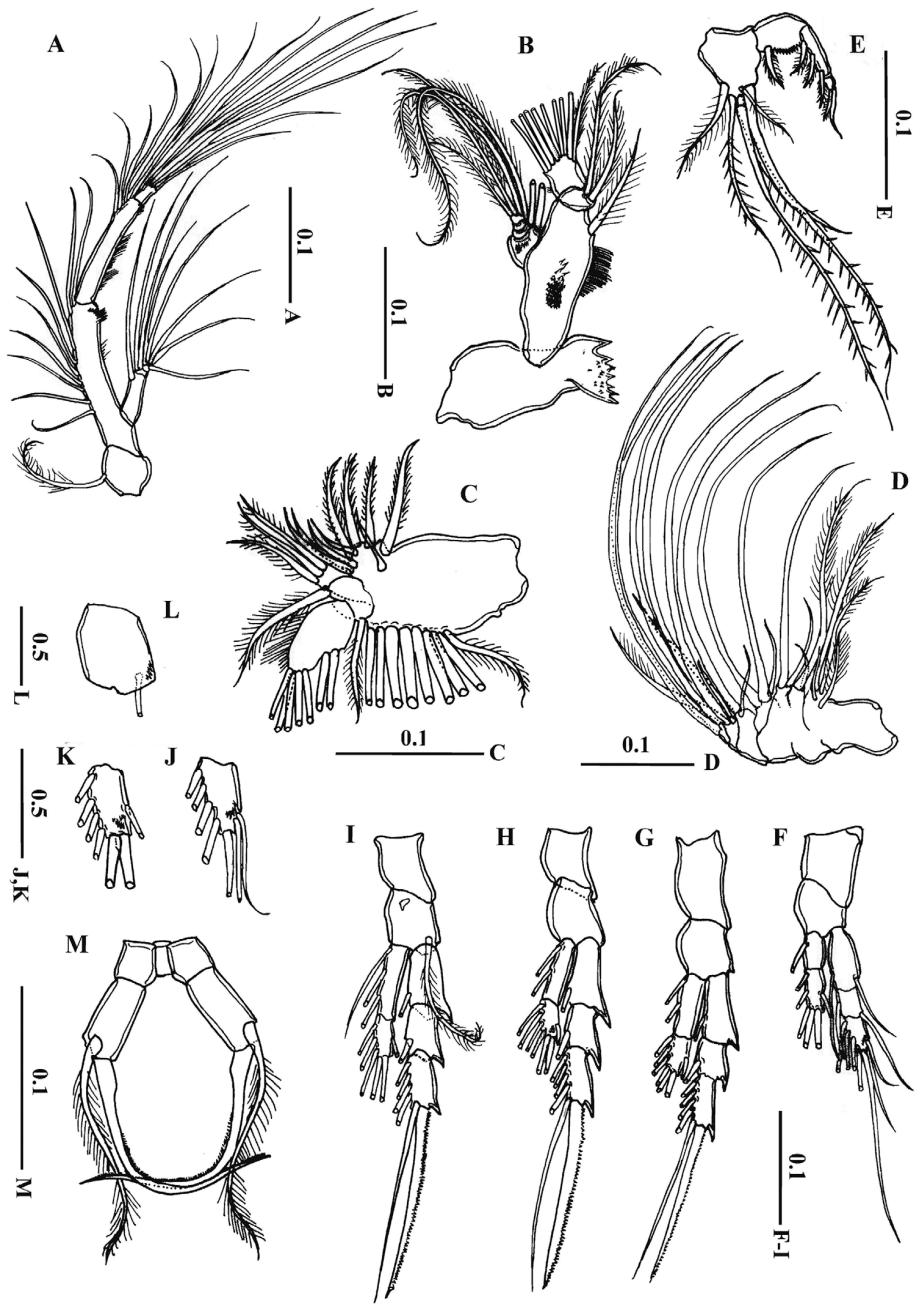
*Acartia bispinosa* female from the northern Red Sea. **A** antenna **B** mandible **C** maxillule **D** maxilla **E** maxilliped **F** Leg 1 **G** leg 2, posterior surface **H** Leg 3, posterior surface **I** leg 4, posterior surface J third exopodal segment of leg 1, anterior surface **K** second endopodal segment of leg 3, anterior surface **L** basis of leg 4, anterior surface **I** leg 5 anterior surface. All scale bars in mm.

Antenna (Fig. 3A) long; coxa with one seta; basis and first endopodal segment completely fused forming elongate allobasis carrying 9 setae along medial margin and oblique row of tiny spinules on distal part of anterior surface; exopod short, 4-segmented with setal formula of 1, 2, 2, 3. Second (first free) endopodal segment elongated bearing 7 setae and furnished with hairs along lateral margin; third (second free) endopodal segment short bearing 7 setae.

Mandible (Fig. 3B): coxa with well-developed gnathobase; basis with 1 medial seta and group of fine spinules posteriorly; exopod 5-segmented, with setal formula of 1, 1, 1, 1, 2; endopod 2- segments, with 2 and 9 setae on first and second segment respectively.

Maxillule (Fig. 3C): praecoxa and coxa incompletely fused; praecoxal arthrite (endite) with 9 setae; coxal endite with 3 setae, coxal exite bearing 9 setae; basis with thick medial seta and short lateral seta; exopod 1-segmented and bearing 2 setae laterally and 5 setae terminally; endopod absent.

Maxilla (Fig. 3D): precoxa and coxa incompletely fused; with 4 endites, each carrying short seta on each and 3, 1, 1, 2 long setae, respectively; basis with 1 long seta and 1 short seta; endopod 4-segmented, with 5 long setae, 2 medium setae and 1 short seta.

Maxilliped (Fig. 3E) comprising robust syncoxa, basis, and 2-segmented endopod; syncoxal endite with 6 setae (2 short, 2 medium and 2 long); basis bearing 1 short spiniform seta; endopod 2-segments, first segment with 3 medial spine-like setae, second segment tapered at end carrying 2 setae (later one spiniform and elongated at tip).

Swimming legs 1 to 4 (Fig. 3F–I) biramous, with 3-segmented exopod and 2-segmented endopod; third exopodal segment of leg 1 with 4-5 spinules proximally on the ventral surface(Fig. 3J); second endopodal segment of leg 3 with group of tiny spinules proximally on the ventral surface (Fig. 3K); basis of leg 4 with 4 spinules ventrally (Fig. 3L). Seta and spine formula as follows:

**Seta and spine formula:** 

	Coxa	Basis	Exopod segments			Endopod segments	
			1	2	3	1	2
Leg 1	0–0	0–0	1–1;	I-1;	2,I,4	0–1;	1, 3, 2
Leg 2	0–0	0–0	0–1;	0–1;	0,I,5	0–2;	1, 2, 4
Leg 3	0–0	0–0	0–1;	0–1;	0,I,5	0–2;	1, 2, 4
Leg 4	0–0	0–1	0–1;	0–1;	0,I,5	0–3;	1, 2, 3

Leg 5 (Fig. 3M): coxae completely fused to intercoxal sclerite; basis about 1.5 times longer than wide; lateral seta nearly as long as claw-like exopod; exopod reduced and swollen at the base posteriorly and distal two-third furnished medially with very fine spinules (Fig. 2D).

**Figure 4.**
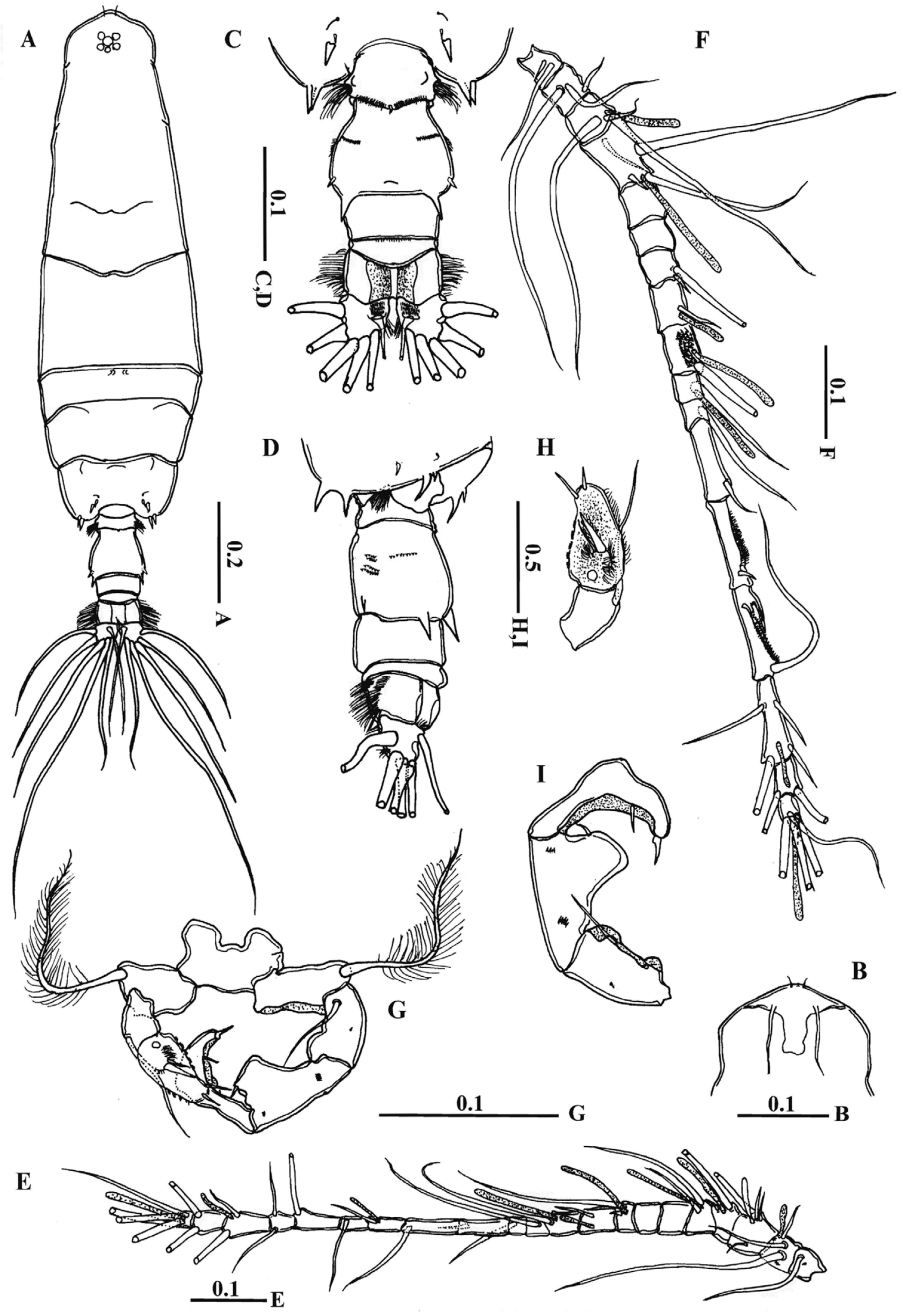
*Acartia bispinosa* male from the northern Red Sea. **A** habitus, dorsal view **B** rostrum, ventral view **C** urosome, dorsal view **D** urosome, latero-ventra view **E** left antennule **F** right antennule **G** leg 5 **H** terminal segment of left exopod of leg 5 **I** terminal segment of right exopod of leg 5. All scale bars in mm.

**Figure 5.**
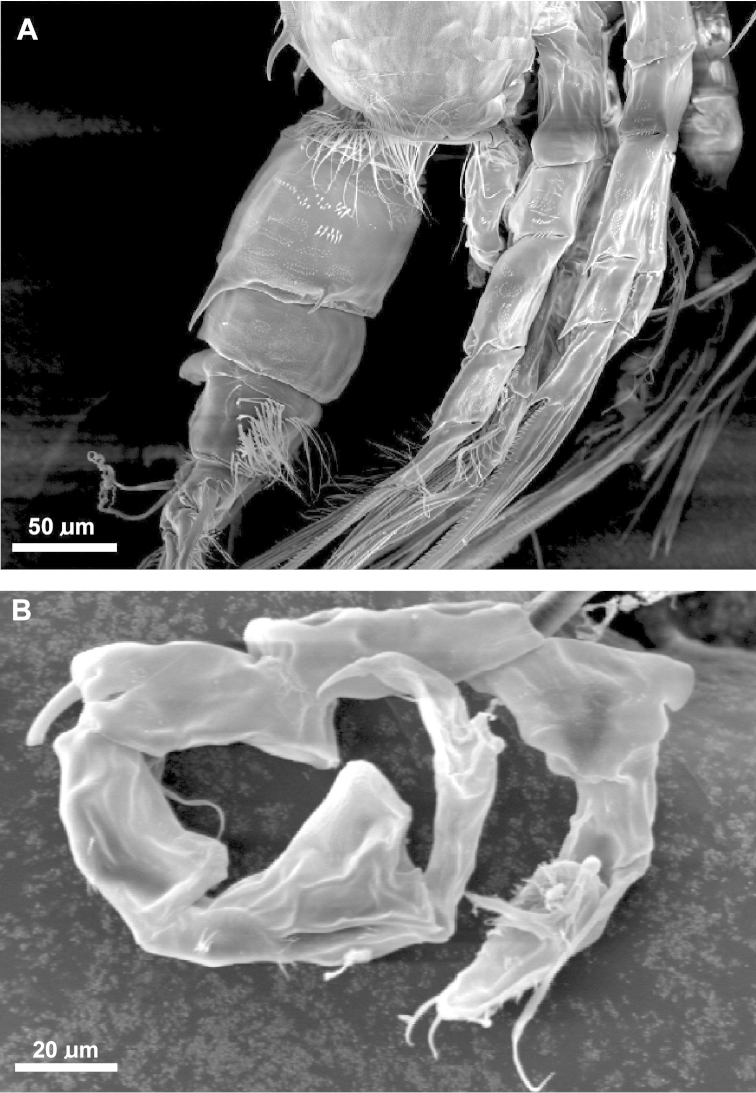
SEM micrographs of *Acartia bispinosa* male from the northern Red Sea. **A** abdomen, lateral view **B** leg 5, posterior surface.

Male: Body (Fig. 4A) slender; rostral filaments long and thin (Fig. 4B). Fifth pediger with 2 subequal pointed posterior processes, smaller than in female, 2 dorsolateral spines and fine setules on each side (Fig. 4C, D). First urosomite with rows of soft hairs on lateral and posterior margin (Fig. 4C, D). Second urosomite with 2 strong dorsolateral spines on posterior margin reaching slightly more than two-thirds of the following somite, 2 ventrolateral small spines, 2 dorsolateral groups of fine spinules ventrolaterally on both sides and dorsolateral row of fine setules along posterior margin (Figs 4C, D, 5A). Third and fourth urosomites with very fine setules along posterior margins. Anal somite bearing short hairs on lateral surface. Caudal ramus with transverse rows of fine setules at the base of medial dorsal seta and with hairs along lateral margin and along distal half of medial margin.

Left antennule (Fig. 4E) 21-segmented; segmentation and setation patterns as follows: (1) I=1, (2) II–IV=3, (3) V–IX=4+ae, (4) X=2+ae, (5) XI=2+ae, (6) XII=unarmed, (7) XIII=unarmed, (8) XIV=2 (1 spiniform)+ae, (9) XV=1+ae, (10) XVI=1+ae, (11) XVII=1, (12) XVIII=1, (13) XIX=1, (14) XX=1, (15) XXI=1+ae, (16) XXII=1, (17) XXIII=1, (18) XXIV=1+1, (19) XXV=1+1+ae, (20) XXVI=1+1, (21) XXVII–XXVIII=4+ae.

Right antennule (Fig. 4F) 17-segmented, with geniculation between thirteenth and fourteen segments; second to fourth segments partly fused on dorsal surface; segmentation and setation patterns as follows: (1) I=2, (2) II–VI=3, (3) VII–VIII=3+ae, (4) IX–XI=4 (1 spiniform)+ae, (5) XII=unarmed, (6) XIII=unarmed,(7) XIV=2 (1 spiniform)+ae, (8) XV=1+ae, (9) XVI=1+ae, (10) XVII=1, (11) XVIII=1+ae, (12) XIX=1, (13) XX=1, (14) XXI–XXIII=2+process, (15) XXIV–XXV=2+2+ae, (16)XXVI=1+1, (17) XXVII–XXVIII=4+ae. Ninth segment covered with tiny spinules on the dorsal surface.

Other mouthparts and leg 1 to leg 4 as in female. Male leg 5 (Figs 4G, 5B) asymmetrical; intercoxal sclerite completely fused to both coxae. Left leg 5 basis nearly two times longer than wide and armed with long later seta; exopod 2-segmented; proximal exopodal segment shorter than distal exopodal segment carrying one seta distally; distal exopodal segment carrying 2 terminal spines, a stout spine at mid-anterior surface with fine setae near its base, 5 groups of very tiny spinules along medial margin, and several spinules along lateral margin (Fig. 4H). Right leg 5 comprising basis armed with lateral seta and 3-segmented exopod; first exopodal segment with 1 long seta; second exopodal segment with distal projection carrying 1 small spine medially and 2 groups of fine spinules on the posterior surface; third exopodal segment armed with terminal stout spine and 1 spine medially near distal end (Fig. 4I).

### Variations

**Figure 6.**
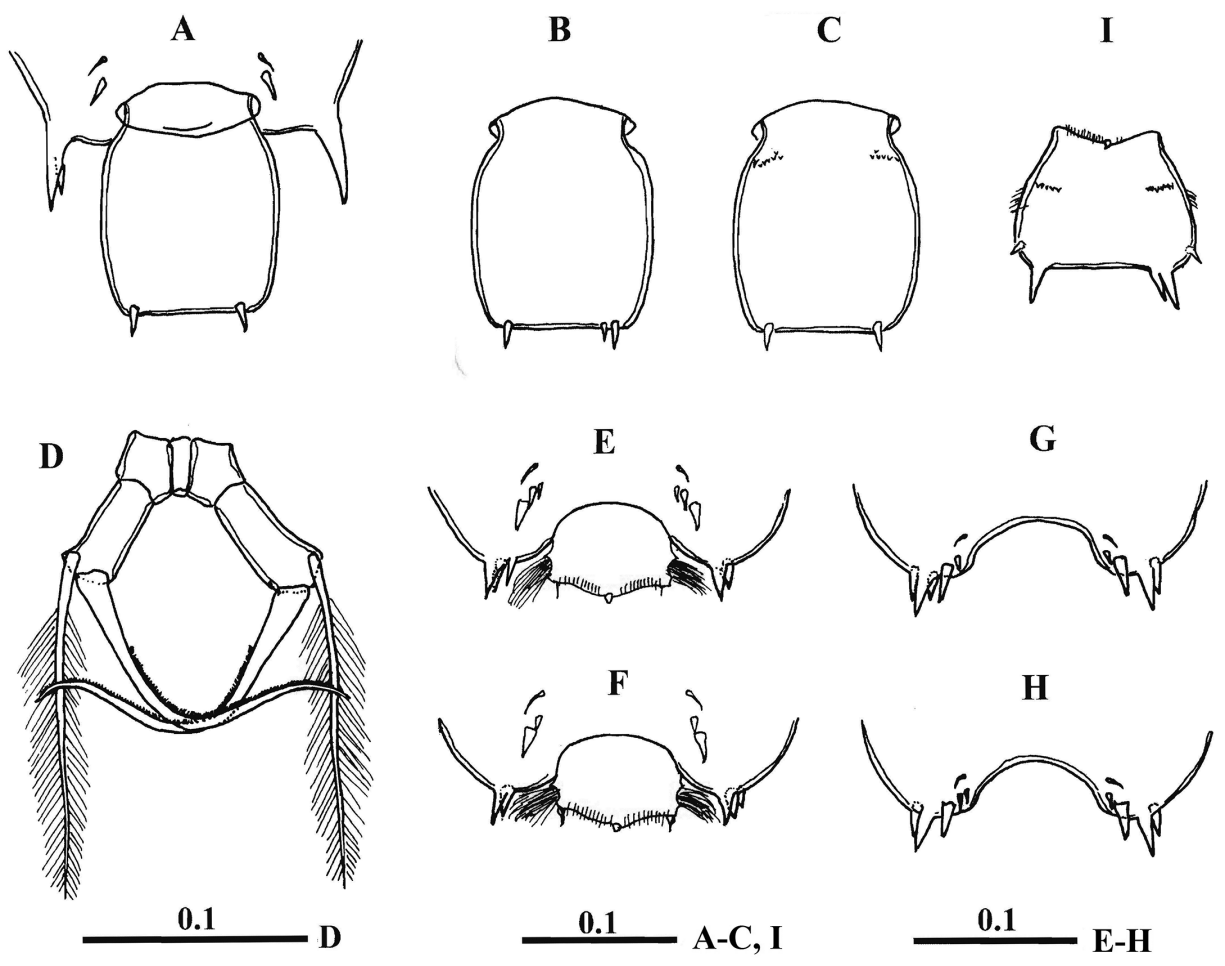
Variations in *Acartia bispinosa* from the northern Red Sea. **A–C** female genital compound somite **D** female leg 5 **E–H** male last pediger **I** male second urosomite. All scale bars in mm.

*Acartia bispinosa* showed some variations in both sexes. In female left projection of prosome can be bifurcated (Fig. 6A), genital compound somite sometimes bearing 2 posterodorsal spines on right side (Fig. 6B), genital compound somite with row of fine spinules anterodorsally on both sides (Fig. 6C), and the degree of leg 5 exopod curvature also varied (Fig. 6D). In male, the lateral projections are with three spines on the posterior corner of fifth pediger (Fig. 6E, F). Also, the number and position of posterodorsally spines of the last pediger varied within species (Fig. 6F–H). Second urosomite sometimes with 2 spines on the right locus (Fig. 6I).

### Seasonal patterns of environmental factors, abundance and body size

**Figure 7.**
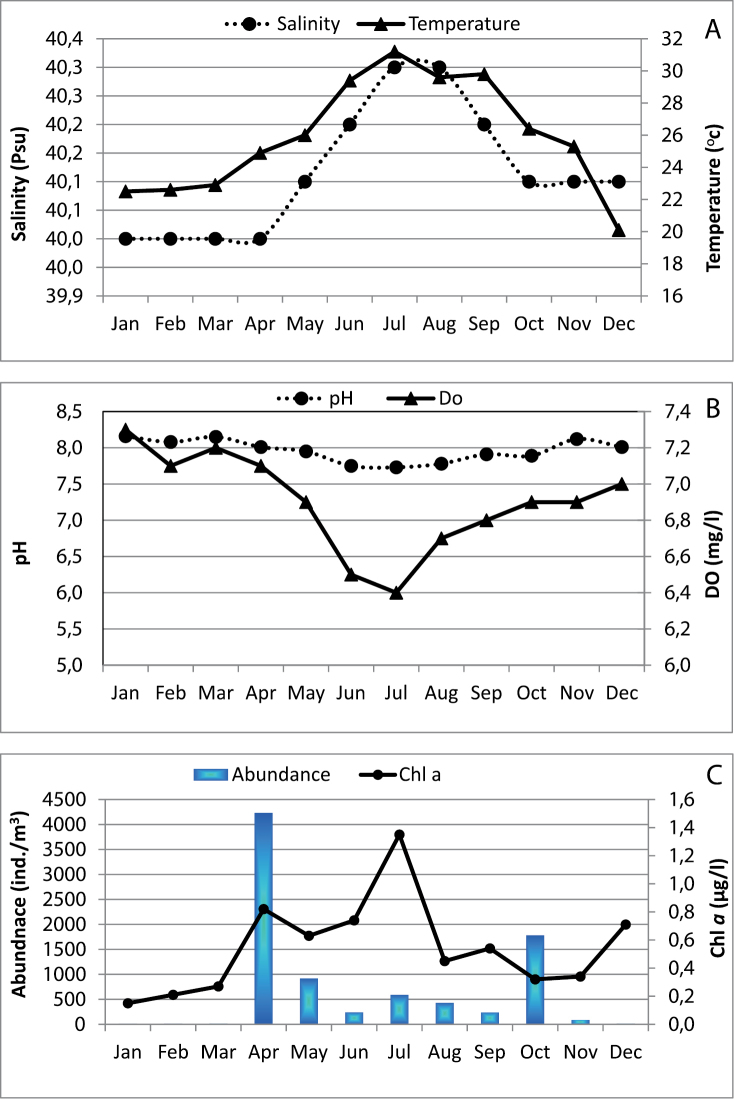
Seasonal patterns at Sharm El-Maya Bay, the northern Red Sea. **A** Temperature and Salinity **B** pH and dissolved oxygen concentrations (DO), and **C** Chlorophyll *a* concentration (chl *a*) and abundance of *Acartia bispinosa*.

Temperature, salinity and chlorophyll *a* measurements over the 12 months investigation are presented in Figure 7. Temperature shows a cyclical annual pattern with the highest value recorded in the end of July (31.2 °C), and the minimum at the end of December (20.1 °C). Salinity was fairly stable (Fig. 7A), showing slight variation between 40 psu in the colder period and 40.3 psu in July-August (annual average: 40.1 psu). Hydrogen ion and dissolved oxygen concentrations showed nearly the same pattern varying between minima of 7.73 and 6.4 mg/l in July and maxima of 8.16 and 7.3 mg/l in January respectively (Fig. 7B). Chlorophyll *a* showed an annual average of 0.54 µg l^-1^ with the maximum recorded in July (1.35 µg l^-1^) with a small increase of 0.82 µg l^-1^ in April (Fig. 7C).

**Figure 8.**
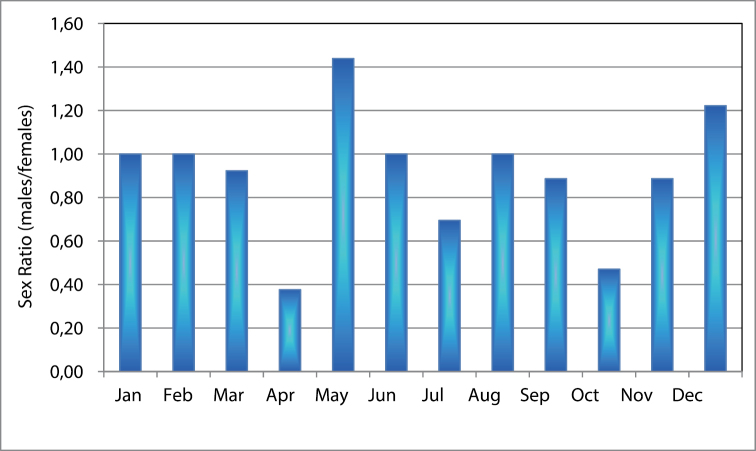
Seasonal variation in males/females sex ratio in the copepod *Acartia bispinosa* in the study area.

Adults of *Acartia bispinosa* were present in the plankton samples throughout the year in our study area with an annual average of 716 individuals m^-3^, forming 12.7% of total zooplankton community. Their abundance pattern showed two peaks (Fig. 7C), a more pronounced one occurred at the end of April (4234 individuals m^-3^, constituting 84% of total zooplankton community) and the second peak was in November (1784 individuals m^-3^). During December-March, very low numbers were found in the samples (not more than 12 individuals m^-3^). Statistically, no significant correlation was observed between the abundance and temperature as well as between the former and chlorophyll *a* concentration (*P* > 0.05, r = 0.065 and 0.288 respectively). In this study, the sex ratio (males/females) of *Acartia bispinosa* showed a clear variation pattern with higher proportion of females observed during the end of April (Spring) and end of October (Autumn) as compared to early summer (end of May and June). The males ratio increased only at the end of May and December forming 60 and 55% of the adult density (Fig. 8).

The minimum, maximum, mean, standard error and standard deviation of total length as well as the prosome length of *Acartia bispinosa* are given in Table 1. In females the highest body size in terms of total length (1.57 mm) and prosome length (1.34 mm) appeared in January and the lowest of 1.20 and 1.02 mm were observed at the end of June. The highest total and prosome length of the male (1.19 and 0.99 mm) were observed in March respectively (Table 1). On the other hand, their lowest value appeared in June.

**Table 1.** Minimum, maximum, mean, standard error (SE) and standard deviation (SD) in total and prosome length of *Acartia bispinosa* sampled in the study area.

Sex	Length (mm)	Minimum (mm)	Maximum (mm)	Mean (mm)	SE (mm)	SD (mm)
**Female**	Total	1.20 (June)	1.57 (Jan.)	1.42	0.017	0.059
	Prosome	1.02 (June)	1.34 (Jan.)	1.16	0.020	0.071
**Male**	Total	1.04 (June)	1.19 (Jan.)	1.12	0.007	0.026
	Prosome	0.83 (June)	0.99 (Mar.)	0.88	0.008	0.028

**Figure 9.**
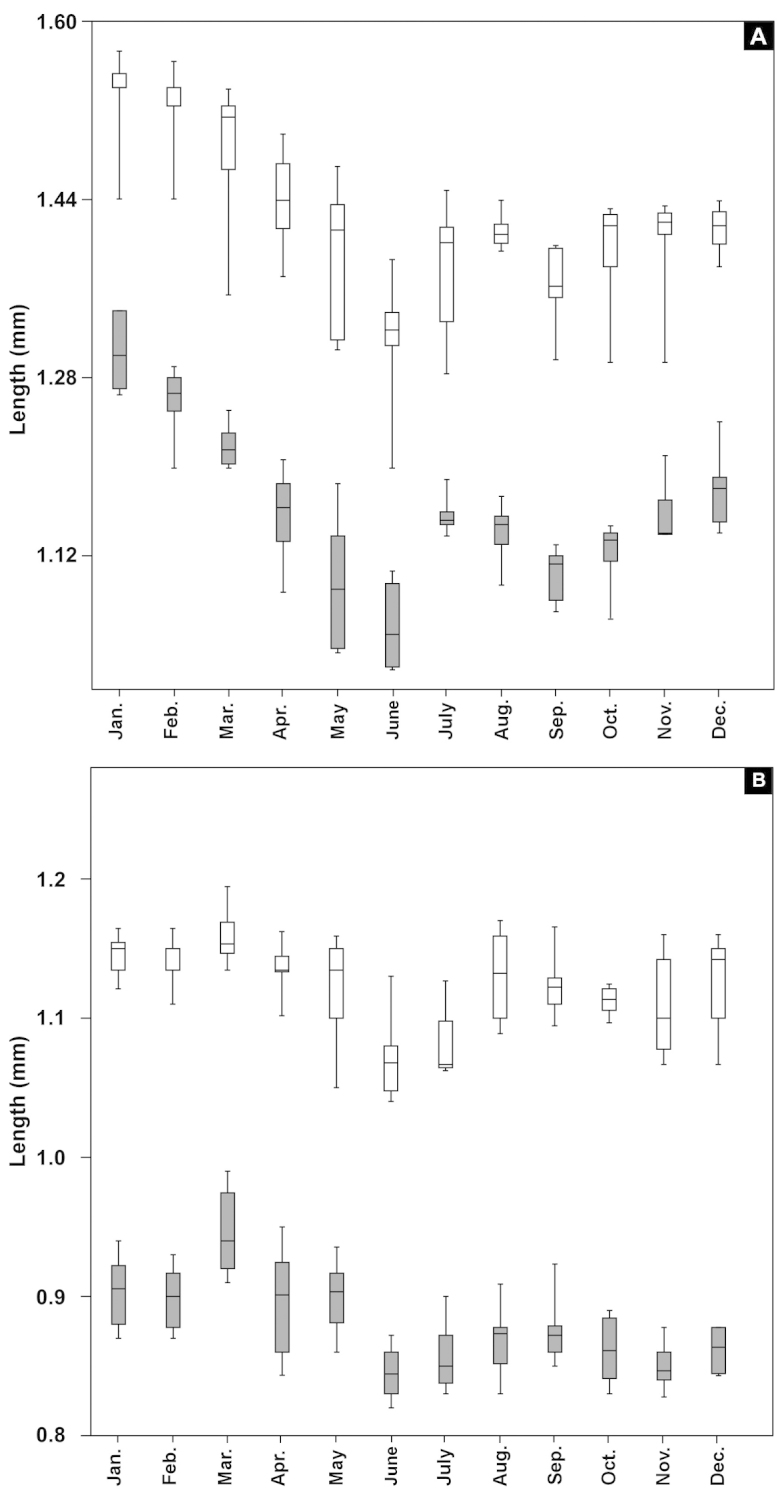
Box and whisker plot of seasonal variations of total and prosome length of female **A** and male **B** of *Acartia bispinosa* (total length: open symbol, prosome length: filled symbol).

In Figure 9, box and whisker plots for total and prosome length of both sexes are shown depicting the median value within a box defined by the interquartile range, and the whiskers representing the range. The total body and prosome length of female decreased from a maximum in January to a minimum at the end of June with a slight increase in late summer and early winter (Fig. 9A). In males, the pattern of variation in total and prosome length showed the maximum in late winter-early spring and minimum in June (Fig. 9B). Also mean values total and prosome length of *Acartia bispinosa* showed nearly the same pattern with minima in June for both sexes and maxima in January for female and in March for male.

Results of analysis of variance (ANOVA) showed significant differences between months for both female and male total length (F= 36.97, *P* < 0.000 for female and F= 15.29, *P* < 0.000 for male). Statistically, total and prosome length of females are inversely correlated with temperature (*r*= -0.639 and -0.664 respectively, *P* < 0.05) and positively with pH, salinity and dissolved oxygen (Tables 2, 3). It did not show any significant correlation with chlorophyll *a* concentration (*r*= -0.512 and -0.441 respectively, *P* > 0.05). Concerning the male, total length showed negative correlations with temperature, salinity as well as chlorophyll *a*, and positive with other parameters (Tables 2, 3), while prosome length did not show any relationship with the measured environmental variables.

**Table 2.** Pearson’s correlation coefficient between length measurements of *Acartia bispinosa* and environmental factors in the northern Red Sea.

Sex	Length	Temp.	pH	Salinity	DO	Chl. *a*
**Female**	Total	-0.631 0.028	0.621 0.031	0.658 0.020	0.771 0.003	-0.523 0.081
	Prosome	-0.647 -0.023	0.610 0.035	0.590 0.043	0.704 0.011	-0.461 0.132
**Male**	Total	-0.609 0.036	0.708 0.010	-0.597 0.041	0.851 0.000	-0.677 0.016
	Prosome	-0.333 0.290	0.394 0.205	-0.571 0.052	0.544 0.067	-0.350 0.269

**Table 3.** Regression analysis of mean total and prosome length (mm) of *Acartia bispinosa* against surface water temperature in the northern Red Sea. (TL: Total length, PL: Prosome length and T: Water temperature).

Sex	Temperature regression equation	r^2^	Sign.
**Female**	TL= -0.012 T+1.721 PL= -0.013 T+1.499	0.398 0.419	0.028 0.023
**Male**	TL= -0.005 T+1.241 PL= -0.002T+0.950	0.371 0.111	0.036 0.290

## Discussion

The original descriptions of *Acartia bispinosa* were brief and the drawings incomplete. Some morphological features were probably overlooked or undescribed by the previous authors (Carl 1907, Steuer 1923, Nishida 1985, Mulyadi 2004). Examination of the *Acartia bispinosa* from the Red Sea revealed some shortcoming in earlier descriptions. Newly confirmed features include: (1) the posteroventral margin of female genital compound somite furnished with fine hairs, (2) the female caudal rami carrying a row of setules anteroventrally, (3) the distal two-third of female leg 5 exopod furnished medially with fine spinules, (4) the last metasomal segment of the male has 2 unequal dorsolateral spines on each side. (5) the second urosomite of the male with 2 dorsolateral groups of fine spinules ventrolaterally on both sides and dorsolateral row of fine setules along posterior margin, (6) the third and fourth urosomites with very fine setules along posterior margins, and (7) the second exopodal segment of the male right leg 5 carrying 2 groups of fine spinules on the posterior surface. This species was more precisely illustrated from the Japanese waters by Nishida (1985) than in the original description. In his paper the above stated feature (4) was already mentioned. The antennules, mouth parts and legs 1–4 of this species are first illustrated and describe herein, according to the conventions of Huys and Boxshall (1991).

In genus *Acartia*, there are currently 62 valid species (Razouls et al. 2014) provisionally divided into seven subgenera: *Acartia* Dana, 1846, *Acartiura* Steuer, 1915, *Euacartia* Steuer, 1915, *Hypoacartia* Steuer, 1915, *Acanthacartia* Steuer, 1915, *Odontacartia* Steuer, 1915, and *Planktacartia* Steuer, 1915. The Acartia subgenus Odontacartia includes twelve species (*Acartia amboinensis* Carl, 1907, *Acartia australis* Farran, 1936, *Acartia bispinosa*, *Acartia bowmani* Abraham, 1976, *Acartia centrura*, *Acartia erythraea*, *Acartia japonica* Mori, 1940, *Acartia lilljeborgii* Giesbrecht, 1889, *Acartia mertoni* Steuer, 1917, *Acartia ohtsukai* Ueda & Bucklin, 2006, *Acartia pacifica* Steuer, 1915, and *Acartia spinicauda* Giesbrecht, 1889) which is categorized into 3 species group as proposed by Steuer (1923) and Ueda and Bucklin (2006), i.e. *erythraea* group (*Acartia erythraea*, *Acartia amboinensis*, *Acartia australis*, *Acartia bispinosa*, *Acartia japonica*), the *centrura* group (*Acartia centrura*, *Acartia spinicauda*) and the *pacifica* group (*Acartia pacifica*, *Acartia mertoni* and *Acartia ohtsukai*) and two intermediate species, *Acartia bowmani* and *Acartia lilljeborgii*.

*Acartia bispinosa* closely resembles *Acartia amboinensis* and *Acartia erythraea*, but it differs from the latter two species in the following characteristics in the female: (1) the second segment of antennule with strong claw-like spine curved proximally from midposterior margin, (2) the exopod of the female leg 5 reduced and swollen at the base posteriorly and distal two-thirds furnished medially with very fine spinules; in the male: (1) left leg 5 distal segment with 2 terminal spines, a stout spine on the mid-anterior surface with fine setae near its base and tuft of hairs proximally, row of spinules along medial margin, and several spinules along lateral margin.

We report this particular species from the Red Sea for the first time. There are three possible explanations of this discovery: 1) most of the plankton studies in the Red Sea were focused mainly in oceanic regions resulting in ruling out of this species which were dominant mainly in the neritic waters, 2) it may have beeen present but overlooked or misidentified in the previous studies and 3) it may be a representative of an invasive species transported in the Red Sea by human activities (possibly in ballast water). In a way we can conclude that the presence of this species in the Red Sea is obviously due to the overlooking by previous authors (e.g. Khalil and Abd El-Rahman 1997, Aamer et al. 2007), due to the resemblance of this species with *Acartia amboinensis*, *Acartia centrura* and *Acartia erythraea*. Also, it can be considered as a normal extension or distribution of the species since most of the Red Sea fauna is Indo-West Pacific in origin.

**Figure 10.**
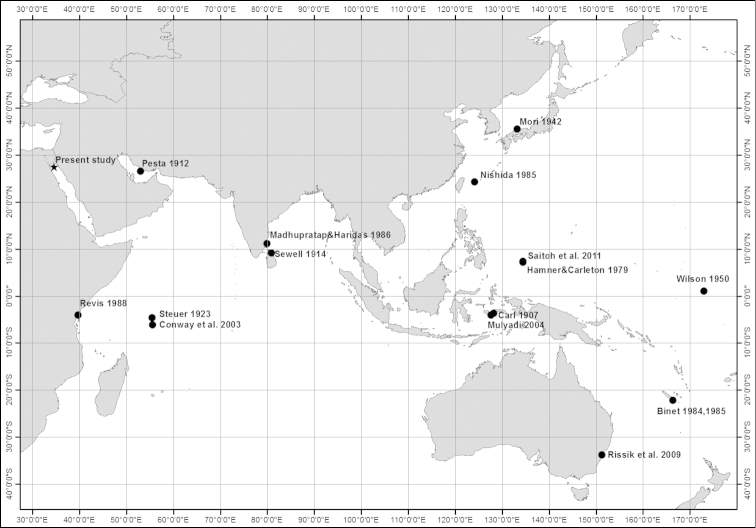
Distribution of *Acartia bispinosa* based on previous records and on the present study (Note that its distribution is restricted between 35°N and 32°S).

*Acartia bispinosa* is distributed mainly in the Indo-West Pacific region. It has been recorded from Ambon Bay, Malaysian coastal waters (Carl 1907, Mulyadi 2004), Persian Gulf (Pesta 1912), coastal water of Sri-Lanka (Sewell 1914), neritic waters of Seychelles, south western Indian Ocean (Steuer 1923, Conway et al. 2003), Tudor Creek, Kenya (Revis 1988), Butaritari lagoon, Gilbert Island and the Fiji Island (Wilson 1950 as *Acartia hamata*), Great Barrier Reef of Palau (Hamner and Carleton 1979, Saitoh et al. 2011), Dee Why Lagoon, Sydney, Australia (Rissik et al. 2009), New Caledonia, South Pacific Ocean (Binet 1984, 1985), Katae Bay (Simane Peninsula) and Kabira Bay (Ishigaki Island, Japan) (Mori 1942 as *Acartia tokiokai*, Nishida 1985 respectively), coastal waters of western Bay of Bengal, northern Indian Ocean (Madhupratap and Haridas 1986). From the distribution pattern, it is evident that this particular species is recorded mainly from tropical and subtropical neritic waters and lagoons which are inhabited with coral reefs and mangrove forests with an average temperature more than 20 °C which is quite similar to the coral reef distribution patterns in these areas (Fig. 10).

The seasonal distribution patterns of *Acartia bispinosa* clearly showed a pronounced peak at the end of April (temperature: 24.9 °C) and a smaller one at the end of October (temperature: 26.4 °C). This pattern is similar to those found in a tidal mangrove-fringing reef lagoon in Kenya, western Indian Ocean (Revis 1988). In his study, Revis (1988) reported the presence of *Acartia bispinosa* in the plankton samples from December to July with prominent peaks during March-April and also in December with a somewhat similar temperature of the present investigation. Also, Tafe and Griffiths (1983) during their studies in Port Hacking, New South Wales, Australia pointed out the disappearance of *Acartia bispinosa* from the plankton samples during June-August and its further re-emergence in higher numbers during March-April (4736 ind. m^-3^) when the temperature was in a range between 20.5–22.6 °C. This may suggest that 20–28 °C is the optimal temperature for maximum production of *Acartia bispinosa*.

Monthly variations in the abundance of *Acartia bispinosa* did not show correlation with any of the measured environmental parameters (temperature, pH, salinity, dissolved oxygen and chlorophyll *a* concentration). This implies that water temperature alone could not explain the variation in the abundance of *Acartia bispinosa*, although water temperature as well as food quantity and quality has been suggested as key parameters influencing the abundance of many *Acartia* species (Dam et al. 1994, Pagano and Saint-Jean 1994, Jonasdottir 1994, Jonasdottir and Kiorboe 1996, Gubanova 2000). In the present study, despite the high chlorophyll *a* concentration in summer due to outbreak of the diatom, *Hemiaulus hauckii* Grunow ex Van Heurck, 1882 and the cyanobacteria, *Trichodesmium* spp., no subsequent significant changes in the abundance or body sizes were noticed. This may be due to the particular food preference of *Acartia bispinosa* and further non usage of the available food matter effectively. Also, the current species is well known for its omnivorous behavior (Tafe and Griffiths 1983), which revealed the importance of other non-algal food items (bacteria, ciliates and detritus). Moreover, the quality of the food may be more important rather than its high concentration to assure optimal growth (Klein Breteler et al. 1990, Hart and Santer 1994).

The character of changes in the population dynamics of *Acartia bispinosa* as well as the very low densities of this species in the plankton from December to March allow us to conclude that the dominance of adult females in April and October, during the condition of gradual warming and cooling of coastal water, is linked to resting eggs, that are capable to give a new generation. Correspondingly, resting eggs of *Acartia* were found in bottom sediment of the different bays all over the world (Lee and McAlice 1979, Liang and Uye 1996). Some authors (Uye and Kasahara 1979, Uye 1980, Onoue et al. 2004) emphasize the importance of such eggs of neritic copepods for maintaining their population potential in the bays and bights through unfavorable seasons.

Seasonal variations in the body sizes have often been observed in marine invertebrates including copepods. In our study, the total length of *Acartia bispinosa* varies less than 51.9% and 41.2% around the annual means for both the females and males respectively. The observed seasonal variability of the body size, seems to be inversely related to temperature, as confirmed by the Pearson correlation. Similar seasonal variability in size with a winter maximum are commonly observed in copepods (Deevey 1960, Uye et al. 1982, Liang and Uye 1996, Belmonte and Cavallo 1997, Mauchline 1998, Riccardi and Mariotto 2000, Ara 2002, Gaudy and Verriopoulos 2004, Tellioglu 2006, Bozkurt and Can 2014). Among the other factors affecting copepod body size, food availability seems to be of great importance, at least in some ecosystems (Deevey 1960, Durbin et al. 1983, Viitasalo et al. 1995). According to Deevey (1960) the seasonal temperature range determines its relative importance along with food in affecting the copepod body size: when the annual mean range was 14 °C or more. In the present study, the temperature range was 10.1 °C and this may explain the weak correlations between *Acartia* abundance with that of temperature and chlorophyll *a* concentration.

Variations in prosomal ends, genital somite and leg 5 of both sexes are common within other species of *Acartia* as reported in many previous works (e.g. Ueda 1986, Garmew et al. 1994, Hirst and Castro-Longoria 1998, Soh and Suh 2000, Ueda and Bucklin 2006).

In the present study, *Acartia bispinosa* has been observed as a monospecific aggregation in the study area during daytime at the end of April. In tropical reef environments many acartiid copepod species exhibit swarms. For example, Acartia (Acanthacartia) spinata Esterly, 1911 and *Acarita tonsa* Dana, 1849 were reported by Emery (1968) on Caribbean reef and *Acartia australis* and *Acartia bispinosa*, were observed in aggregations on Great Barrier Reef (Hamner and Carleton 1979). Similarly, swarms of *Acartia hamata* were found in the fringing coral reefs of Sesoko island, southern Japan (Ueda et al. 1983) as well as *Acartia* swarms in Conch Reef off Florida (Heidelberg et al. 2010). Most of the swarming of this species appeared as monospecific aggregations, which is a common phenomenon in copepods (Ueda et al. 1983, Ambler et al. 1991). According to many authors (Hamner and Carleton 1979, Ueda et al. 1983, Ambler et al. 1991, Buskey 1998), the advantages of swarming behavior in copepods are: (1) protection against predation, (2) reduction of dispersion by currents, (3) facilitating and enhancing mating opportunity, and (4) keeping in good position to feed on coral mucus.

## Conclusion

In this study we have reported the presence of *Acartia bispinosa* in the Red Sea for the first time with full redescription. There are three possible explanations of this discovery: 1) most of the plankton studies in the Red Sea were focused mainly in oceanic regions resulting in ruling out of this species which were dominant mainly in the neritic waters, 2) it may have be present but overlooked or misidentified in the previous studies and 3) it may be a representative of an invasive species transported in the Red Sea anthropogenically (possibly in ballast water). The seasonal distribution patterns of *Acartia bispinosa* clearly showed a pronounced peak at the end of April and a smaller one at the end of October during the condition of gradual warming and cooling of coastal water, that may be linked to resting eggs, that are capable to give a new generation. Females showed their highest total and prosome length in January and the lowest were observed at the end of June. The highest total and prosome length of the male were detected in March and the lowest value appeared in June. This variability of the body size, seems to be inversely related to temperature, indicating the influence of other environmental parameters.

## Supplementary Material

XML Treatment for Acartia (Odontacartia) bispinosa
